# Bioinformatics Prediction and Experimental Validation Identify a Novel Cuproptosis-Related Gene Signature in Human Synovial Inflammation during Osteoarthritis Progression

**DOI:** 10.3390/biom13010127

**Published:** 2023-01-07

**Authors:** Wenjuan Wang, Ziyi Chen, Yinghui Hua

**Affiliations:** Department of Sports Medicine, Huashan Hospital, Fudan University, No. 12, Wulumuqi Zhong Road, Shanghai 200040, China

**Keywords:** cuproptosis, immune infiltration, osteoarthritis

## Abstract

Osteoarthritis (OA) is the one of most common joint diseases worldwide. Cuproptosis, which had been discovered lately, is a novel form of cell death induced by copper. Our purpose is to study the relationship between cuproptosis-related genes (CRGs) and inflammatory microenvironments in patients with OA and identify characteristic cuproptosis-related biomarkers. First, the combinatory analysis of OA transcriptome data from five datasets identified differentially expressed CRGs associated with OA. Then, we applied single-sample gene set enrichment analysis (ssGSEA) to evaluate immune-cell infiltration and immune-function levels in OA patients and normal controls, respectively. Hub CRGs for OA were mined based on the random forest (RF) model, and a nomogram prediction model was constructed based on them. In total, four differentially expressed CRGs were identified through bioinformatics analysis and confirmed by RT-qPCR. *FDX1* and *LIPT1* were expressed at a high level in OA, while *DBT* and *DLST* were expressed higher in the normal group. In total, 10 CRGs were found to be significantly correlated with immune landscape. Four hub CRGs were subsequently obtained by the RF analysis as potential biomarkers for OA. We constructed an OA predictive model based on these four CRGs (*DBT*, *DLST*, *FDX1,* and *LIPT1*).

## 1. Introduction

Osteoarthritis (OA) is a common joint disorder that exerts an extensive health burden on the affected individuals, health-care systems, and wider socioeconomic costs [1,2,3]. The occurrence of OA is related to multiple elements such as inflammation, senescence, fatness, joint injuries, metabolic disorders, and so on [2]. Pathological changes in OA involve almost all of the joint tissues, including cartilage, subchondral bone, the synovial membrane, etc. [4]. Clinical diagnosis based on symptoms (pain, brief morning stiffness, and functional limitations) and a brief physical examination are the golden criteria for confirming OA [2]. The typical management of OA is palliative and reactive, and joint-replacement surgery can be performed when appropriate [2]. As the exact pathogenesis of OA is still unclear currently, there are no effective drugs to slow down the progression of the disease [5].

As the boundary between the internal structure of the joint and adjacent soft tissues, the synovial membrane is very important to maintain the stability of the joint microenvironment. Although in osteoarthritic joints the primary histopathological change is cartilage destruction, synovitis is also a common feature of OA [4]. It has been reported that the release of inflammatory factors and degrading enzymes in the synovial tissues is closely related to the severity of OA [4]. Therefore, synovitis is a participant in OA progression rather than a bystander, suggesting that synovitis may be a key potential target for OA therapy.

Research has shown that the advancement of OA is regulated by multiple different mechanisms of cell death, including pyroptosis, ferroptosis, apoptosis, and necroptosis [6]. In a recent study published in Science, Tsvetkov et al. discovered a new form of copper-dependent cell death termed cuproptosis [7]. This is nonapoptotic cell death that involves intracellular copper accumulation, mitochondrial lipoylated proteins aggregation, and Fe–S cluster proteins destabilization [7,8,9,10,11]. In OA joints, mitochondrial function in chondrocytes and synoviocytes is severely disturbed, characterized by enhanced inflammation, increased apoptosis, augmented catabolic activity, and decreased mitochondrial biogenesis [12,13]. The hypoxia state of synovial tissue alters the response of synovial cells to apoptotic stimuli [12]. Additionally, Yazar et al. found that in synovial fluid, Cu and Fe is concentrated more densely in patients with OA than in healthy subjects (*p* < 0.05) [14]. Therefore, it would be reasonable to speculate that in OA progression cuproptosis may play an important role. However, the potential regulatory mechanisms of cuproptosis in OA have yet to be elucidated and require further exploration.

In our study, for the first time, we systematically investigated the differentially expressed cuproptosis-related genes (CRGs) and immune characteristics between normal and OA individuals. We applied the support vector machine (SVM) learning and random forest (RF) methods to identify the cuproptosis-related key biomarkers and used the drug database to obtain the cuproptosis-related ideal drug targets for OA.

## 2. Materials and Methods

### 2.1. Data Source and Differentially Expressed Genes Acquirement

The transcriptome profiles and clinical information of 38 OA patients and 36 healthy controls were downloaded from five datasets (GSE1919, GSE41038, GSE55235, GSE82107, and GSE55457) on the gene-expression omnibus (GEO) (http://www.ncbi.nlm.nih.gov/geo/, assessed on 14 August 2022) database. We then employed the SVA method to merge the GSE1919, GSE41038, GSE55235, GSE82107, and GSE55457 datasets [15].

CRGs were extracted from previous literature by Tsvetkov et al. [7]. Finally, 11 CRGs were included in our research, containing *FDX1*, *LIPT1*, *LIAS*, *DLD*, *DBT*, *DLST*, *DLAT*, *PDHA1*, *PDHB*, *ATP7A,* and *ATP7B*.

Differentially expressed CRGs between OA patients and normal controls were screened out by applying the “limma” package, with the criteria setting as *p* < 0.05 and a |log fold change (FC)| >1 [16]. The visualization of the chromosomal localization of CRGs was accomplished using the R circos package [17].

### 2.2. Immune Infiltration Analysis

Given that immune-cell infiltration such as T cells, B cells, and macrophages was detected in synovial tissues of OA patients [18], we further comprehensively investigated the total immune landscape, which included both immune-cell infiltration and immune function using the single sample gene set enrichment analysis (ssGSEA) algorithm. The ssGSEA method was applied to evaluate the abundance of 17 immune-cell infiltration and 13 immune-function levels in the OA patients and normal controls [19]. Spearman’s correlation analysis was performed between CRGs and the immune infiltration using the R ggcorrplot package [20].

### 2.3. The Hub Genes Were Screened Based on the RF Analysis

To predict the occurrence of OA, we constructed a training model adopting both the SVM and RF methods. Boxplots of residuals, the reverse cumulative distribution of residuals, and the receiver operating characteristic (ROC) curve were used to compare the accuracy of the two models. The RF method was then selected to screen differentially expressed CRGs using the R library ‘randomForest’ with ‘mtry’ and ‘ntree’ setting to 3 and 500, respectively [21]. The optimal ‘ntree’ was chosen according to minimum cross-validation error in 10-fold cross-validation, and the significance of differentially expressed CRGs with the optimal ntree was assessed. We then constructed a nomogram using the ‘rms’ package [22,23]. Calibration curves were used to evaluate the consistency between the observed and predicted values. Finally, we performed clinical-impact-curve and decision-curve analyses to evaluate the clinical benefits of our model.

### 2.4. Functional Enrichment Analysis of 4 Cuproptosis Hub Genes

The biological process (BP), cellular component (CC), and molecular function (MF) of gene ontology (GO), and the Kyoto Encyclopedia of Genes and Genomes (KEGG) enrichment analysis of 4 CRGs, were performed on Enrichr (https://maayanlab.cloud/Enrichr/, assessed on 14 August 2022) [24], an interactive and collaborative gene-list-enrichment-analysis tool. The *p* value < 0.05 was considered significantly enriched.

### 2.5. Protein–Protein Interaction (PPI) Network Analysis

In order to portray functional and physical interactions among cuproptosis in OA utilizing the STRING (https://string-db.org/, assessed on 14 August 2022) (version 11.0) repository, we respectively constructed the PPI network of proteins derived from all CRGs included in our study and four hub CRGs [25]. In this experiment, the low confidence value was set as 0.15 to generate the PPI network of CRGs.

### 2.6. Recognition of Transcription Factors and miRNAs Engage with 4 Hub Genes

NetworkAnalyst is a broad online platform for the meta-analysis of gene expression data [26]; JASPAR is a publicly available resource for TFs profiles for multiple species [27]; and MirTarbase is the one of biggest experimental validity databases for miRNAs–target gene interactions [28]. We have utilized the JASPAR database on the NetworkAnalyst platform to figure out topologically credible TFs that tend to bind to our hub genes. MiRNAs that interact with hub genes focused on topological analysis were extracted from the interaction of miRNA–gene on mirTarbase database via networkAnalyst.

### 2.7. Identification of Potential Small Molecules for OA

The drug signatures database (DSigDB) on the Enricher platform was used to generate the small molecules that could downregulate the expression of hub genes [24,29].

### 2.8. Sample Collection

Synovial tissue from 3 patients of meniscus injury and 3 of OA were collected from Huashan hospital. All patients critically read and signed the informed consent form (KY2020-060), which was approved by the ethics committee of Huashan Hospital. The research followed the guidelines of the 1975 Declaration of Helsinki.

### 2.9. Reverse-Transcription Quantitative Polymerase Chain Reaction (RT-qPCR)

The total synovial tissue RNA was extracted using Trizol (Servicebio), and then total RNA was reverse-transcribed to complementary DNA (cDNA) using Servicebio^®^RT Enzyme Mix. The qRT-PCR was performed using the 2×SYBR Green qPCR Master Mix (None ROX) (Servicebio). The primer sequence of genes used in our study is listed in Table S1. Genes were normalized to GAPDH. Relative levels of mRNA were expressed as fold-changes as calculated by the 2^−ΔΔCT^ method. Each biological sample was technically performed in triplicate.

### 2.10. Statistical Analysis

All statistical analyses in our study were performed with R software, version 4.1.1. For all figures: * represents *p* < 0.05, ** represents *p* < 0.01, and *** represents *p* < 0.001.

## 3. Results

### 3.1. Differentially Expressed CRGs in OA

**Figure 1:**
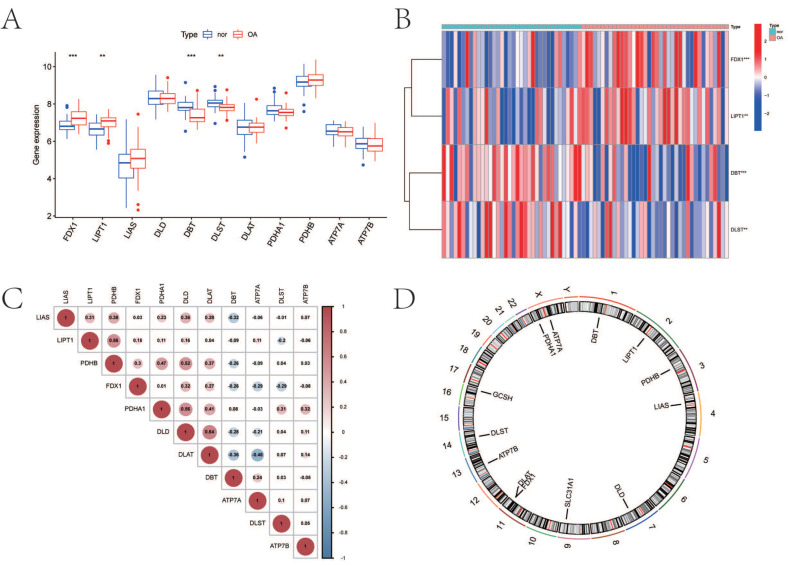
Expression characteristics and gene localization of CRGs. (**A**) Boxplot showing differences in the expression of CRGs in OA and normal tissues, with significant differences in the expression of 4 genes. (**B**) Heat map showing the expression characteristics of CRGs in OA tissues and normal tissues. Red showing high expression levels while blue showing low expression levels. (**C**) Spearman correlation analysis of CRGs; positive correlation is represented by red, while negative correlation is represented by blue. (**D**) The position of CRGs on the chromosome (for all figures: ** represents *p* < 0.01, and *** represents *p* < 0.001). CRGs, cuproptosis-related genes; OA, osteoarthritis.

We extracted the gene expression matrix of 11 CRGs from patients with OA (*n* = 38) and normal subjects (*n* = 36). The distribution of differentially expressed CRGs between normal controls and OA patients was visualized by a boxplot and a heatmap in Figure 1A,B. It showed that *FDX1* (*p* < 0.001) and *LIPT1* (*p* < 0.01) were expressed more highly in OA, while *DBT* (*p* < 0.001) and *DLST* (*p* < 0.01) were expressed more lowly in OA. The relevant heat map of CRGs was shown in Figure 1C. To illustrate, *DLD* expression was significantly positively correlated with *DLAT* expression, and *DLAT* expression was significantly positively correlated with *ATP7A* expression (Figure 1C). The chromosome location demonstrated that *DBT* was localized on chromosome 1, *LIPT1* on chromosome 2, *PDHB* on chromosome 3, *LIAS* on chromosome 4, *DLD* on chromosome 7, *SLC31A1* on chromosome 9, *DLAT* and *FDX1* on chromosome 11, *ATP7B* on chromosome 13, *DLST* on chromosome 14, *GCSH* on chromosome 16, and *PDHA1* and *ATP7A* on chromosome X (Figure 1D).

### 3.2. Immune Infiltration Analysis

**Figure 2:**
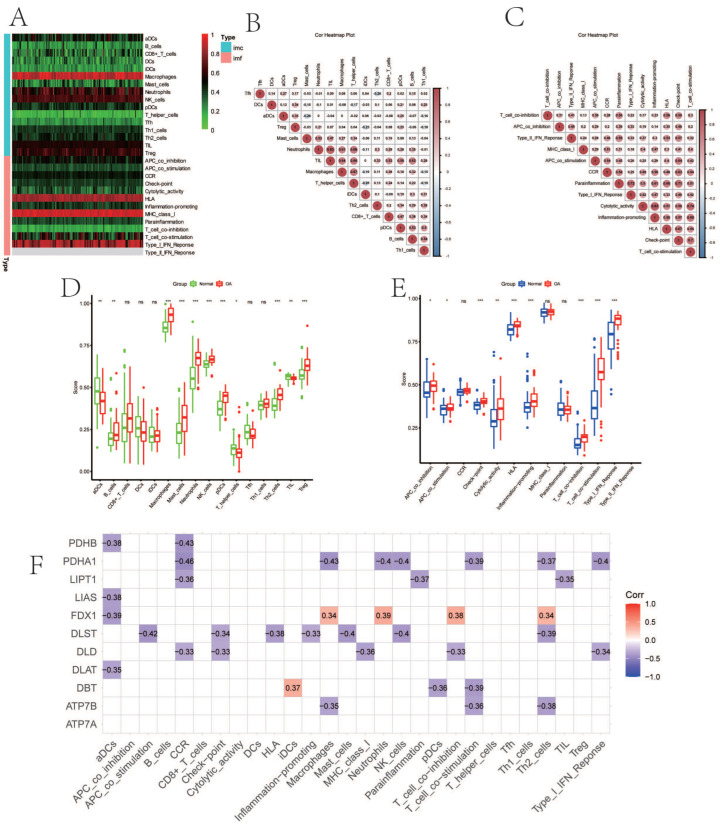
The immune landscape and correlation analysis between CRGs and immune infiltration in OA. (**A**) Heatmap showing the expression characteristics of immune cells and immune function in OA and normal tissues. Red represents high expression levels, while green represents low expression levels. (**B**,**C**) The correlation analysis of immune cells (**B**) and immune function (**C**); positive correlation is represented by red, while negative correlation is represented by blue. (**D**,**E**) Boxplot showing differences in the expression of immune cells (**D**) and immune function (**E**) in OA and normal tissues. (**F**) Correlation between immune infiltration and CRGs; positive correlation is represented by red, while negative correlation is represented by blue. (For all figures: * represents *p* < 0.05, ** represents *p* < 0.01, and *** represents *p* < 0.001). CRGs, cuproptosis-related genes; OA, osteoarthritis.

Figure 2A demonstrated immune-cell infiltration and immune function enrichment in each sample. The relevant heat map of immune cells and immune functions are displayed, respectively (Figure 2B,C). Figure 2D showed that B cells (*p* < 0.01), macrophages (*p* < 0.001), mast cells (*p* < 0.001), neutrophils (*p* < 0.001), natural killer (NK) cells (*p* < 0.001), T helper cells (*p* < 0.001), and tumor-infiltrating lymphocytes (TIL) (*p* < 0.001) expressed higher in OA. Figure 2E indicated that immune function, including antigen-presenting cell (APC) co-inhibition (*p* < 0.05), APC co-stimulation (*p* < 0.05), check-point (*p* < 0.001), cytolytic activity (*p* < 0.01), human leukocyte antigen (HLA) (*p* < 0.001), inflammation-promoting (*p* < 0.001), para-inflammation (*p* < 0.001), T cell co-inhibition (*p* < 0.001), T cell co-stimulation (*p* < 0.001), type I interferon (IFN) response (*p* < 0.001), and type II IFN response (*p* < 0.001) were enriched in OA. The CRGs expression and immune infiltration were correlated (Figure 2F), and 10 genes (*FDX1*, *LIPT1*, *LIAS*, *DLD*, *DBT*, *DLST*, *DLAT*, *PDHA1*, *PDHB,* and *ATP7B*) were mostly related. To illustrate, *PDHA1* expression was significantly negatively correlated with cytokine–cytokine receptor interaction (CCR), while *FDX1* expression was significantly positively correlated with neutrophils (Figure 2F).

### 3.3. The SVM and RF Methods Were Used to Construct an OA Predictive Model Based on Four CRGs

**Figure 3:**
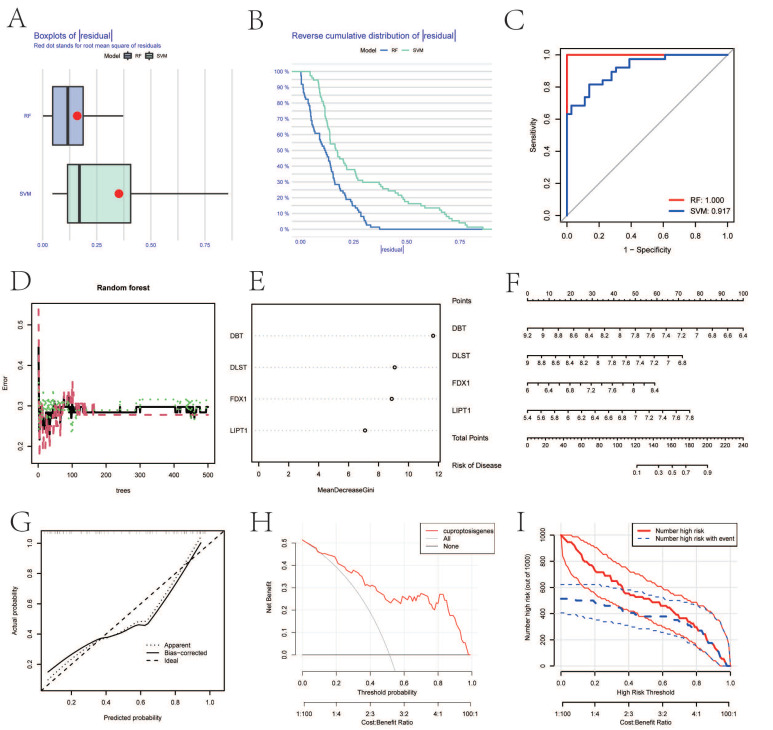
SVM and RF methods were used to screen hub genes. (**A**–**C**) Boxplot of the residual distribution (**A**), reverse cumulative distribution of residuals (**B**), and ROC curves (**C**) as a function of the values of observed sensitivity between RF and SVM. (**D**) RF prediction error curves based on 10-fold cross-validation. (**E**) The importance of the four CRGs based on the RF model. (**F**) Nomogram of the predictive model based on four CRGs. (**G**) Calibration curves showing that the nomogram model may be an ideal predictive model for OA. (**H**,**I**) DCA (**H**) and clinical impact plots (**I**) were used to determine the clinical utility of the risk prediction nomograms. SVM, support vector machine; RF, random forest; ROC, receiver operating characteristic; CRGs, cuproptosis-related genes; OA, osteoarthritis; and DCA, decision-curve analysis.

Boxplots of residuals (Figure 3A), reverse cumulative distribution of residuals (Figure 3B), and ROC curve analysis (Figure 3C) demonstrated that RF displayed notably high predictive capability. According to the minimum cross-validation error in 10-fold cross-validation, the best ‘ntree’ was selected (Figure 3D). In total, we identified four CRGs (*DBT*, *DLST*, *FDX1*, and *LIPT1*) and ranked them according to their importance (Figure 3E). To predict the probability of OA, we constructed a nomogram evaluation mode based on four CRGs (Figure 3F). The calibration curves (Figure 3G), decision-curve analysis (DCA) (Figure 3H), and clinical impact plots (Figure 3I) proved the nomogram model to be an ideal predictive model for OA. 

### 3.4. Function Enrichment Analyses of 4 Hub Genes

**Figure 4:**
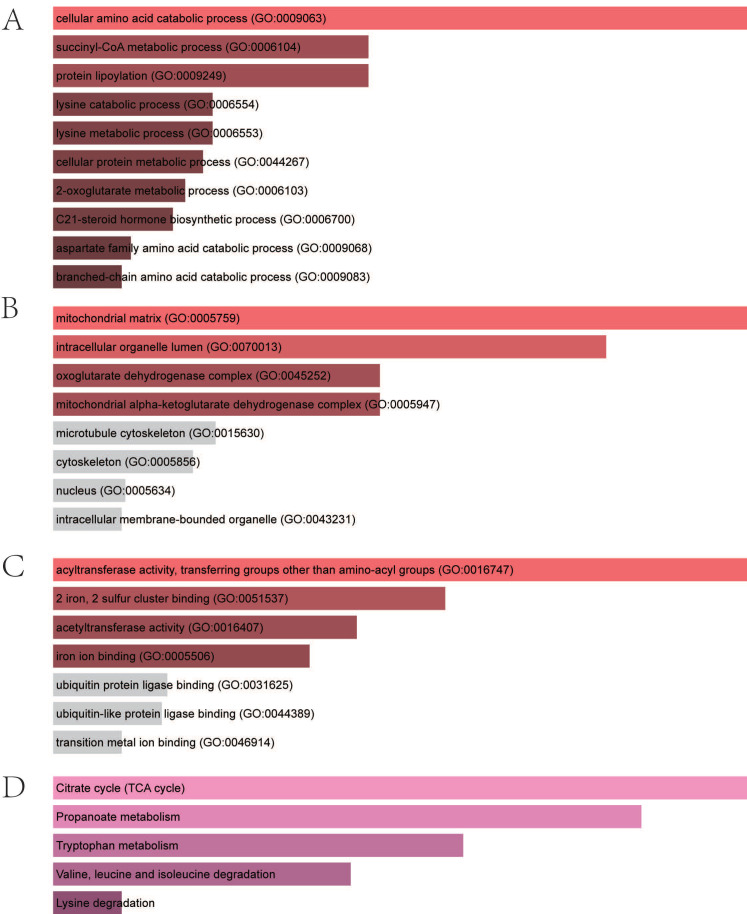
Enrichment analysis of four hub genes. (**A**) Significantly enriched biological processes. (**B**) Significantly enriched cellular components. (**C**) Significantly enriched molecular functions. (**D**) Significantly enriched KEGG pathway. KEGG, Kyoto Encyclopedia of Genes and Genomes.

We performed enrichment analysis to identify distinct biological roles for four CRGs. The BP analysis of GO terms demonstrated that the genes were significantly enriched in the cellular amino acid catabolic process, the succinyl-CoA metabolic process, protein lipoylation, the lysine catabolic process, the lysine metabolic process, the cellular protein metabolic process, the 2-oxoglutarate metabolic process, the C21-steroid hormone biosynthetic process, the aspartate family amino acid catabolic process, and the branched-chain amino acid catabolic process (Figure 4A). With regards to the CC, the genes were mostly related to the mitochondrial matrix, intracellular organelle lumen, the oxoglutarate dehydrogenase complex, and the mitochondrial alpha–ketoglutarate dehydrogenase complex (Figure 4B). For the MF of GO terms, hub genes were mostly related to acyltransferase activity, transferring groups other than amino-acyl groups, two irons, two sulfur-cluster bindings, and acetyltransferase activity and iron ion binding (Figure 4C). The KEGG results exhibited that the genes were mainly related to citrate cycle, propanoate metabolism, tryptophan metabolism, valine, leucine, and isoleucine degradation and lysine degradation (Figure 4D).

### 3.5. Clarification of Protein–Protein Network

**Figure 5:**
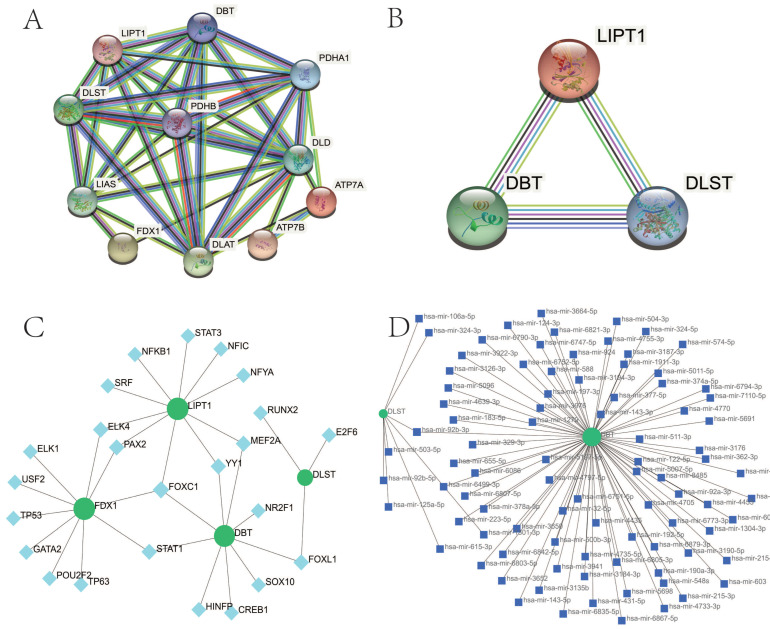
PPI network of CRGs in OA. (**A**) The PPI network of 11 CRGs had 11 nodes and 35 edges. (**B**) The PPI network of four hub CRGs. CRGs, cuproptosis-related genes; PPI, protein–protein interaction; OA, osteoarthritis. (**C**) The cohesive regulatory interaction network of four hub gene–TF. Herein, the square nodes were TFs, and gene symbols interacted with TFs as circle nodes. TF, Transcription factors. (**D**) The interconnected regulatory interaction network of four hub gene–miRNA. Herein, the square node indicated miRNAs and gene symbols interacted with miRNAs as a circle shape. miRNAs, microRNAs.

As depicted in Figure 5A, the PPI network of 11 CRGs consisted of 11 nodes and 35 edges. The PPI network of hub–gene interactions is shown in Figure 5B.

### 3.6. Determination of Regulatory Signatures

TF regulators’ and miRNA regulators’ interaction with four hub CRGs were depicted in Figure 5C,D, respectively. We ascertained 28 TFs and 91 miRNAs regulatory signatures.

### 3.7. Prediction of Candidate Drugs

The top 10 drug candidates associated with CRGs were selected based on *p*-values and adjusted *p*-values. Table 1 showed the top 10 candidate drugs.

### 3.8. Validation of Hub Genes

**Figure 6:**
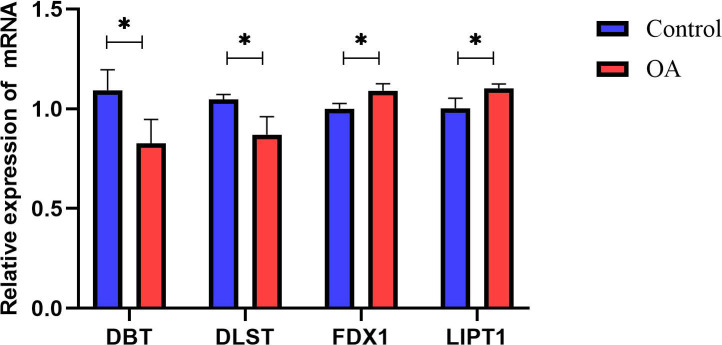
Validation of hub CRGs using qRT-PCR. The relative mRNA expressions of DBT, DLST, FDX1, and LIPT1 were displayed (* represents *p* < 0.05).

We confirmed the four cuproptosis-related biomarkers using RT-qPCR in order to verify our results. In comparison with the control group, the expression of *DBT* and *DLST* were down-regulated in OA synovial tissue; however, the expression of *FDX1* and *LIPT1* were significantly up-regulated (Figure 6). These results were consistent with our predictions using bioinformatics tools.

## 4. Discussion

OA is the most common arthritis, and it is placing an increasing health burden on individuals and society [2]. Due to the heterogeneity of its clinical manifestations and a lack of effective treatment strategies, the underlined pathogenesis of OA needs to be clarified, and a model to exactly predict the risk of OA occurrence is demanded [2,30]. As a recently reported form of copper-dependent cell death, cuproptosis is closely associated with the progression of many diseases [7]. However, its regulatory roles have not been clearly demonstrated, especially in inflammatory diseases [7]. Therefore, we sought to elucidate the specific role of CRGs in the OA phenotype and its association with the immune microenvironment of OA. Our study comprehensively investigated the expression profiles of CRGs in synovial tissues between normal subjects and OA patients, with the upregulation of *FDX1* and *LIPT1* and the downregulation of *DBT* and *DLST* in OA. We then observed whether there was a correlation between CRGs expression levels. These results indicated that CRGs, especially *FDX1*, *LIPT1*, *DBT,* and *DLST*, may be involved in OA development through a regulatory network. We further demonstrated the immune-cell infiltration and immune-function levels in OA using the ssGSEA algorithm. The correlation between CRGs and immune cell infiltration was then calculated in order to clarify the CRG signature in the immune landscape of OA, and most of CRGs were negatively regulated. In order to construct a reliable predictive model, we made a comparison with the performance of the SVM and RF methods and finally obtained four cuproptosis-related biomarkers *(FDX1*, *LIPT1*, *DBT,* and *DLST)* by the RF analysis. RT-qPCR was performed to verify our findings, and it was consistent with results from bioinformatics tools, which reaffirmed the important role of CRGs in OA. We established a cuproptosis nomogram for predicting the risk of OA. Different scores were assigned to *FDX1*, *LIPT1*, *DBT,* and *DLST*. The factor scores were summed up to obtain the total score. If the gross score was no more than 120, the possibility of occurrence of OA was less than 0.1, and if the total score was more than 200, the chance of OA was greater than 0.9.

Four genes (*FDX1*, *LIPT1*, *DBT,* and *DLST)* were determined as hub CRGs in OA. Similar to many other microelements such as iron, copper is a fundamental element playing an important role as a cofactor for essential enzymes that are necessary for human activities [31]. Homeostatic mechanisms maintained the concentration of intracellular copper ions at very low levels, and once the threshold was exceeded, the copper became toxic, directly leading to cell death [32]. Recently, the specific mechanism by which copper triggers cell death, termed cuproptosis, has been elucidated: excess intracellular copper induced the aggregation of specific lipoylated enzymes, which was associated with the mitochondrial tricarboxylic acid (TCA) cycle, resulting in proteotoxic stress and cell death [7]. During this process, scientists identified several key genes: *DBT* (dihydrolipoamide branched chain transacylase E2) and *DLST* (dihydroli-poamide S-succinyltransferase) were among four of the enzymes that were lipoylated by Cu, and they encoded enzymes that regulated carbon entry into the TCA cycle; *FDX1* (Ferredoxin 1, a direct target of copper ionophores) served as an upstream molecule to regulate protein lipoylation; and *LIPT1* (lipolytransferase 1) encoded lipoic acid pathway-related enzymes [7,9]. In our research, the downregulation of *DBT* and *DLST,* together with the upregulation of *FDX1* and *LIPT1,* may indicate the potential role of cuproptosis in OA synovitis.

However, our study still has some limitations that need to be emphasized [6,31,32,33,34]. Firstly, the data source was obtained from a public database, and input errors could not be assessed. Second, it would be better to include more detailed clinical features to confirm the performance of the predictive model. Furthermore, RT-qPCR was performed to verify the different expressions between OA and healthy samples; however, more experiments such as flow cytometry and single-cell sequencing still need to be supplemented to specifically clarify the mechanism.

Overall, we unveiled the correlation between CRGs and immune infiltration in OA patients. A four-CRGs-based RF machine learning model was constructed, which can accurately predict the occurrence risk of OA patients. Our study, for the first time, explored the role of cuproptosis in OA, which may be helpful for elucidating the underlying molecular mechanisms leading to OA progression in the future.

## Figures and Tables

**Table 1:** Suggested top 10 small molecules for OA. OA, osteoarthritis.

TERM	OVERLAP	*p*-VALUE	ADJUSTED *p*-VALUE	ODDS RATIO	COMBINED SCORE	GENES
**LATAMOXEF HL60 DOWN**	3/1578	0.0018453476993364856	0.11186607610867805	35.08761904761905	220.87963432826848	DBT;FDX1;DLST
**ETHOTOIN HL60 DOWN**	1/24	0.004791660011821214	0.11186607610867805	289.463768115942	1545.9907779727164	DBT
**BETULINIC ACID PC3 DOWN**	1/30	0.0059868860535258585	0.11186607610867805	229.50574712643677	1174.6526106132212	DBT
**STAUROSPORINE MCF7 DOWN**	2/649	0.0060396749482483575	0.11186607610867805	29.905718701700156	152.8004312050089	LIPT1;DBT
**CLOPERASTINE PC3 DOWN**	1/37	0.007379956522320185	0.11186607610867805	184.8148148148148	907.2536215941285	DBT
**15-DELTA PROSTAGLANDIN J2 MCF7 DOWN**	1/38	0.00757884707428762	0.11186607610867805	179.8108108108108	877.9072583460236	DLST
**VITINOIN CTD 00007069**	2/780	0.008648746155768222	0.11186607610867805	24.701799485861184	117.3419689357197	DBT;DLST
**EMETINE PC3 UP**	2/801	0.009107965037095087	0.11186607610867805	24.026282853566958	112.89003604044395	LIPT1;DBT
**CAPTOPRIL PC3 DOWN**	2/856	0.010363518033129621	0.11186607610867805	22.414519906323186	102.42233105955586	DBT;DLST
**SULFAMONOMETHOXINE HL60 DOWN**	1/55	0.010955418103578725	0.11186607610867805	123.09876543209876	555.6581197291017	DBT

## Data Availability

The datasets presented in this study can be found in online repositories (GEO, https://www.ncbi.nlm.nih.gov/geo/, assessed on 14 August 2022).

## Supplementary Materials

The following supporting information can be downloaded at: https://www.mdpi.com/article/10.3390/biom13010127/s1, Table S1: Primers employed in this study.

## Abbreviations

OA	Osteoarthritis
CRGs	Cuproptosis-related genes
GEO	Gene-expression omnibus
ssGSEA	Single-sample gene-set enrichment analysis
RF	Random forest
PPI	Protein–protein interaction
RT-qPCR	Real-time quantitative PCR
ROC	Receiver operating characteristic;
BP	Biological process
CC	Cellular component
MF	Molecular function
KEGG	Kyoto Encyclopedia of Genes and Genomes
DSigDB	Drug signatures database
NK	Natural killer
TIL	Tumor-infiltrating lymphocytes
APC	Antigen-presenting cell
HLA	Human leukocyte antigen
IFN	Type I interferon
CCR	Cytokine–cytokine receptor interaction
TCA	Tricarboxylic acid
DBT	Dihydrolipoamide branched chain transacylase E2
DLST	Dihydroli-poamide S-succinyltransferase
FDX1	Ferredoxin 1, a direct target of copper ionophores
LIPT1	Lipolytransferase 1
