# Supplementary material for: Bioinformatics Prediction and Experimental Validation Identify a Novel Cuproptosis-Related Gene Signature in Human Synovial Inflammation during Osteoarthritis Progression
Source: Biomolecules. 2023 Jan 7;13(1):127. doi: 10.3390/biom13010127 (PMC9855951; doi:10.3390/biom13010127)
Supplement: Supplementary file 1 [file biomolecules-13-00127-s001.zip › biomolecules-2066923-supplementary.pdf]

Table S1 Primers employed in this study

Gene symbol	Forward primer (5'-3')	Reverse primer (5'-3')
DBT	TCTTAAAGGCTGCTTCCTTGG	ATCAAACCCTGCTCAGTATCCA
DLST	CTAACAGCAGGAAGGTTGTCATT	CCACCTGACATCTCCCTCTGT
FDX1	CCACTTTATAAACCGTGATGGTG	ACATGCACCAAAGCCATCAA
LIPT1	CTCACCAGGTTCAACAAGCAGTT	CCTACCAATTACAACAGAGGGAGA
